# Exosomes derived from umbilical cord mesenchymal stem cells reduce microglia-mediated neuroinflammation in perinatal brain injury

**DOI:** 10.1186/s13287-019-1207-z

**Published:** 2019-03-21

**Authors:** Gierin Thomi, Daniel Surbek, Valérie Haesler, Marianne Joerger-Messerli, Andreina Schoeberlein

**Affiliations:** 1 Department of Obstetrics and Feto-maternal Medicine, University Women’s Hospital, Inselspital, Bern University Hospital, Bern, Switzerland; 2 Department for BioMedical Research (DBMR), University of Bern, Bern, Switzerland; 3 Graduate School for Cellular and Biomedical Sciences, University of Bern, Bern, Switzerland

**Keywords:** Preterm birth, Perinatal brain damage, White matter injury, Neuroinflammation, Hypoxia-ischemia, Mesenchymal stem cells, Umbilical cord, Extracellular vesicles, Exosomes, Intranasal, Microglia, BV-2

## Abstract

**Background:**

Preterm newborns are at high risk of developing neurodevelopmental deficits caused by neuroinflammation leading to perinatal brain injury. Human Wharton’s jelly mesenchymal stem cells (hWJ-MSC) derived from the umbilical cord have been suggested to reduce neuroinflammation, in part through the release of extracellular vesicle-like exosomes. Here, we studied whether exosomes derived from hWJ-MSC have anti-inflammatory effects on microglia-mediated neuroinflammation in perinatal brain injury.

**Methods:**

Using ultracentrifugation, we isolated exosomes from hWJ-MSC culture supernatants. In an in vitro model of neuroinflammation, we stimulated immortalized BV-2 microglia and primary mixed glial cells with lipopolysaccharide (LPS) in the presence or absence of exosomes. In vivo, we introduced brain damage in 3-day-old rat pups and treated them intranasally with hWJ-MSC-derived exosomes.

**Results:**

hWJ-MSC-derived exosomes dampened the LPS-induced expression of inflammation-related genes by BV-2 microglia and primary mixed glial cells. The secretion of pro-inflammatory cytokines by LPS-stimulated primary mixed glial was inhibited by exosomes as well. Exosomes interfered within the Toll-like receptor 4 signaling of BV-2 microglia, as they prevented the degradation of the NFκB inhibitor IκBα and the phosphorylation of molecules of the mitogen-activated protein kinase family in response to LPS stimulation. Finally, intranasally administered exosomes reached the brain and reduced microglia-mediated neuroinflammation in rats with perinatal brain injury.

**Conclusions:**

Our data suggest that the administration of hWJ-MSC-derived exosomes represents a promising therapy to prevent and treat perinatal brain injury.

**Electronic supplementary material:**

The online version of this article (10.1186/s13287-019-1207-z) contains supplementary material, which is available to authorized users.

## Background

Preterm birth is the leading cause for neonatal morbidity and mortality in developed countries [1]. Survivors of preterm birth are at risk of developing neonatal morbidities such as infections, necrotizing enterocolitis, bronchopulmonary dysplasia, and brain injury [2, 3].

Perinatal brain injury causes typical neurodevelopment deficits such as motor impairments, mental and developmental retardation, learning disabilities, and psychiatric disorders later in life [4–6]*.* The pathogenesis of perinatal brain injury is complex, but is thought to involve both inflammation and ischemia leading to the formation of free radicals and subsequent death of neurons and pre-oligodendrocytes [7]. Additionally, the innate immune response plays a key role in the pathogenesis of perinatal brain injury. The main mediators of the innate immune response to brain injury are microglial cells, the brain’s resident macrophages. Once activated upon injury, microglial cells release a large number of inflammatory factors designed to limit infectious processes. However, this immune defense mechanism causes additional brain injury and contributes substantially to the subsequent neurodevelopment deficits [8]. Hence, multiple studies have shown that therapies targeting microglia-mediated inflammation confer neuroprotection in several types of brain injuries [9–12], suggesting that microglia may be a novel therapeutic target for perinatal brain injury [13]*.*

Stem cell-based therapies have recently been shown to be an effective treatment for some of the main morbidities associated with preterm birth [14]. Mesenchymal stem cells (MSC) have been shown to be promising therapeutic candidates for preterm birth-associated diseases due to their widely known regenerative and immunomodulatory capacities [15–17].

Human Wharton’s jelly mesenchymal stem cells (hWJ-MSC) are particularly promising for treating preterm-related bronchopulmonary dysplasia [18] and amniotic fluid mesenchymal stem cells for treating severe necrotizing enterocolitis [19]. With regards to perinatal brain injury, hWJ-MSC have also been shown to reduce neuroinflammation and induce neuroregeneration [14]. Although hWJ-MSC express neural markers and are able to differentiate into neuroglia cells in vitro [20–22], their regenerative effects are thought to be primarily exerted via modulation of the host immune response [23]. Accordingly, numerous studies have shown that MSC secrete anti-inflammatory cytokines [12, 24] and release extracellular vesicles [10] such as exosomes [9, 25] that modulate the immune response.

Exosomes are small membrane vesicles (30–150 nm in diameter) secreted during the fusion of multivesicular endosomes with the plasma membrane [26]. They mediate intercellular communication by transferring cell surface receptors, cytokines, lipids, mRNAs, microRNAs, and long noncoding RNAs from their cell of origin to their target cell [26]. MSC-derived exosomes hold great potential for cell-free therapies because they offer safety and low immunogenicity while still exhibiting the same immunomodulatory and regenerative capacities of their mother cells [27]. MSC-derived extracellular vesicles have been shown to inhibit microglia activation [10], and exosomes have been used therapeutically to reduce neuroinflammation after traumatic brain injury [9]. Furthermore, MSC-derived exosomes exhibit particular promise for treating preterm-associated diseases such as severe necrotizing enterocolitis [28] and bronchopulmonary dysplasia [29]. Therefore, our goal was to explore the anti-inflammatory potential of exosomes derived from hWJ-MSC for the treatment of perinatal brain injury.

## Methods

### Isolation and culture of human Wharton’s jelly-derived mesenchymal stem cells (hWJ-MSC)

MSC were isolated from the connective tissue of the human umbilical cord known as Wharton’s jelly. Umbilical cords from six different donors were collected with written informed consent from women with uncomplicated pregnancies undergoing elective cesarean sections at term (mean gestational age 38 ± 1.8 weeks, *n* = 6). hWJ-MSCs were isolated from the umbilical cords via enzymatic digestion as previously described [30] and cultured in expansion medium consisting of Dulbecco’s modified Eagle’s medium (DMEM)/F12 supplemented with 10% fetal bovine serum (FBS), 2 mmol/l GlutaMAX™, 100 units/ml penicillin, and 100 μg/ml streptomycin (Thermo Fisher Scientific, Waltham, MA, USA).

### Characterization of hWJ-MSC

At passage number 6, hWJ-MSCs were visualized with bright field microscopy followed by flow cytometric analysis of cell surface markers. The cells were stained with fluorescein isothiocyanate-conjugated mouse monoclonal antibodies against human cluster of differentiation (CD) 105 (AbD Serotec, Oxford, UK), CD90 (Acris Antibodies, San Diego, CA, USA), CD73 (BD Biosciences, Franklin Lakes, NJ, USA), CD45 (BD Biosciences), CD34 (BD Biosciences), CD19 (Millipore, Billerica, MA, USA) and CD14 (Millipore), and human leukocyte antigen–antigen D related (HLA-DR) (BD Biosciences). An Alexa-Fluor 594-conjugated anti-mouse IgG antibody (Thermo Fisher Scientific) was used to detect the unconjugated mouse monoclonal antibodies against human CD73 (BD Biosciences) and CD19 (Millipore). Antibodies were diluted in 1% FBS and phosphate-buffered saline (PBS) to their working concentration and incubated with hWJ-MSC for 15 min at 4 °C. At least 10′000 events were acquired on a LSR II flow cytometer (BD Biosciences) and data were analyzed using the FlowJo software (Tree Star, Inc., Ashland, OR, USA).

### Isolation of hWJ-MSC-derived exosomes

Exosomes were isolated from WJ-MSC culture supernatants using serial centrifugations according to the protocol of Théry et al. [31] and as we previously described [32]. The pelleted exosomes were resuspended in PBS and stored at − 20 °C.

### Characterization of hWJ-MSC-derived exosomes

Exosomes were characterized according to their size and surface marker expression using negative-staining electron microscopy and an Exo-Check Exosome Antibody Array.

#### Negative-staining electron microscopy

The size and shape of hWJ-MSC-derived exosomes were analyzed with electron microscopy. Aliquots of 5 μl exosomes were adsorbed on Formvar® (Formvar resin 15/95, Ted Pella, Inc., Redding, CA, USA) coated copper grids for 30 s, washed three times with pure water, and negatively stained with 2% uranyl acetate solution (Electron Microscopy Sciences, Hatfield, PA, USA) for 30 s. Excess fluid was removed by gently pushing the copper grids sideways to filter paper. Samples were visualized with a transmission electron microscope (CM12, Philips, Eindhoven, Netherlands) equipped with a digital camera (Morada, Soft Imaging System, Münster, Germany) and image analysis software (iTEM; OSIS, Olympus Soft Imaging Solutions, Münster, Germany).

#### Exo-check exosome antibody array

The exosomal surface marker expression of hWJ-MSC-derived exosomes was confirmed using the Exo-Check Exosome Antibody Array (System Biosciences, Palo Alto, CA, USA). Exosomes were incubated on a membrane with 12 pre-printed spots of antibodies against exosomal markers CD63, CD81, ALG-2-interacting protein X (ALIX), flotillin 1 (FLOT1), intercellular adhesion molecule 1 (ICAM1), epithelial cell adhesion molecule (EpCAM), annexin A5 (ANXA5), and tumor susceptibility gene 101 (TSG101). The GM130 cis-Golgi protein marker served as a control to exclude cellular contamination. Human serum exosome proteins served as positive controls. The array was performed according to the manufacturer’s instruction.

### BV-2 microglia culture

The semi-adherent mouse cell line BV-2 (ATL03001) was purchased from Banca Biologica e Cell Factory, Genoa, Italy, and expanded in Roswell Park Memorial Institute (RPMI) 1640 with 10% FBS, 2 mmol/l GlutaMAX™, 100 units/ml penicillin, and 100 mg/ml streptomycin. BV-2 cells were detached from culture plates by mechanical vibrations and flushing with PBS.

### Mixed glial cell culture

Primary mixed glial cells were isolated from cortexes of 2-day-old Wistar rat pups according to the protocol of Chen et al. [33]. Mixed glial cells were seeded at a density of 36′000 cells/cm^2^cultured in DMEM supplemented with 10% FBS, 2 mmol/l GlutaMAX™, 1 mmol/l sodium pyruvate, 100 units/ml penicillin, and 100 μg/ml streptomycin (Thermo Fisher Scientific) on poly-d-lysine (100 μg/ml, Sigma-Aldrich) coated dishes.

### Stimulation of BV-2 and mixed glial cells with lipopolysaccharide (LPS) and co-culture with hWJ-MSC-derived exosomes

BV-2 and mixed glial cells were seeded at a density of 18'000 cells/cm^2^ and 36'000 cells/cm^2^, respectively, before being incubated with 100 ng/ml LPS (Sigma-Aldrich) for 0, 15, 30, or 60 min for intracellular signaling analysis (BV-2 cells only), for 6 h for inflammation-related gene expression and cytokine analysis and for 24 h for cytokine secretion analysis. hWJ-MSC-derived exosomes (1 μg/ml) were added simultaneously with LPS. As controls, cells were cultured either without the addition of LPS and exosomes or with 1 μg/ml exosomes only.

### Animal model of perinatal brain injury

**Fig. 1:**
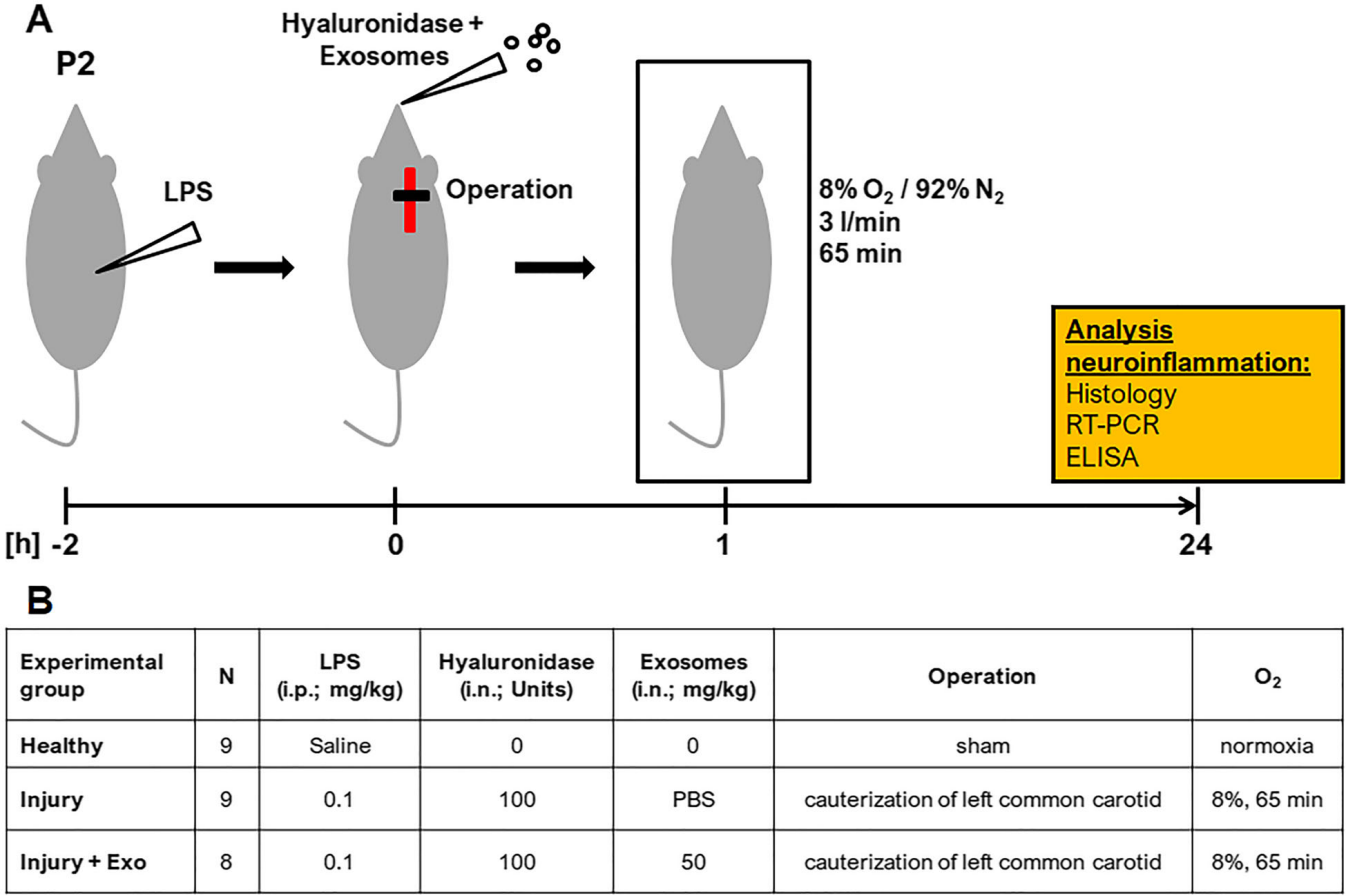
Rat model of perinatal brain injury. **a** Schematic representation of the experimental outline. Perinatal brain injury was induced in postnatal day 2 (P2) rat pups by i.p. injection of LPS, followed by left common carotid artery cauterization 2 h later and exposure to hypoxia for 65 min. Hyaluronidase was administered into both nostrils 30 min before i.n. exosome or PBS application and the latter was done immediately before the cauterization of the left common carotid. **b** Detailed overview of the three experimental groups. *ELISA enzyme-linked immunosorbent assay*, *Exo* exosomes, *i.p.* intraperitoneal, *i.n.* intranasal, *n* number of animals, *P2* postnatal day 2, *RT-PCR* reverse transcription polymerase chain reaction

Perinatal brain injury was introduced in 2-day-old Wistar rat pups using a combination of hypoxic-ischemic and inflammatory insults outlined in Fig. 1a. For this, pregnant Wistar rats (Janvier Labs, Le Genest-Saint-Isle, France) were housed under specific pathogen-free conditions on a 12-h light/dark cycle with ad libitum access to water and standard laboratory chow. On postnatal day 2, rat pups were randomly assigned to three experimental groups (Fig. 1b): Healthy (*n* = 9), Injury (*n* = 9), and Injury + Exo (*n* = 8). Healthy animals received a saline injection, were sham operated, kept under normoxic conditions, and received no exosomes. Injury and Injury + Exo animals received an intraperitoneal injection of 0.1 mg/kg LPS in saline (*Escherichia coli* 0111:B4; Sigma-Aldrich), followed by the cauterization of the left common carotid artery 2 h later and exposure to hypoxia (8% O_2_/92% N_2_, 3 l/min,) for 65 min, as previously described [14]. Between the LPS injection and the ligation, Injury + Exo animals received exosomes in PBS (50 mg/kg) by intranasal administration, whereas Injury animals received PBS only. An increased permeability of the nasal mucosa was ensured by a 1 μl drop of hyaluronidase (100 U in PBS, Sigma-Aldrich) into the nostril 30 min before the exosome or PBS administration. For inflammation-related gene and cytokine expression, Healthy (*n* = 5), Injury (*n* = 6), and Injury + Exo (*n* = 5) animals were sacrificed 24 h post insult on postnatal day 3 by decapitation. Brains were collected and stored at − 80 °C. For immunohistochemistry, Healthy (*n* = 4), Injury (*n* = 3), and Injury + Exo (*n* = 3) animals were sacrificed with sodium pentothal and transcardially perfused with PBS and 4% paraformaldehyde 24 h post insult on postnatal day 3. Brains were fixed in 4% paraformaldehyde and embedded in paraffin.

### Exosome uptake into BV-2 and mixed glial cells

#### Confocal microscopy

Exosomes were stained with 2 × 10^−6^ M PKH26 according to the manufacturer’s protocol (Sigma-Aldrich). BV-2 and mixed glial cells were seeded at a density of 25,000 cells/cm^2^ and 50,000 cells/cm^2^, respectively, in chamber slides for overnight attachment before they were co-cultured with PKH26-labeled exosomes for 6 h. Co-cultures were then fixed with 4% paraformaldehyde and blocked with 1% bovine serum albumin (BSA; Sigma-Aldrich) and 0.25% Triton X-100 (Sigma-Aldrich) in PBS for 1 h at room temperature. Cells were stained overnight with a rabbit primary antibody against β-tubulin (1:200, ab6046, Abcam, Cambridge, UK) at 4 °C followed by the detection with an anti-rabbit IgG Alexa Fluor 488 secondary antibody (1:200, Thermo Fisher Scientific) at room temperature for 1 h. Nuclei were counterstained using 4′,6-diamidino-2′-phenylindole-dihydrochloride (DAPI; Sigma-Aldrich). Confocal images were acquired on a laser scanning microscope (Carl Zeiss LSM 710) with a 63x magnification. Images were processed in Imaris software licensed to the Microscopy Imaging Center of the University of Bern.

#### Flow cytometry

Exosomes were stained with 2 × 10^−6^ M PKH26. PKH26-labeled exosomes (1 μg/ml) were cultured with BV-2 (25′000 cells/cm^2^) and mixed glial cells (50′000 cells/cm^2^) in 10-cm cell culture dishes for 15 min, 30 min, 3 h, 6 h, or 8 h. After co-culture, cells were harvested and fixed with 1% paraformaldehyde. At least 10'000 events were acquired on a LSR II flow cytometer (BD Biosciences), and data were analyzed using the FlowJo software (Tree Star, Inc).

### RNA and protein isolation

RNA and protein were isolated using the QIAshredder and the Allprep DNA/RNA/Protein Mini Kit according to the manufacturer’s protocol (Qiagen, Hilden, Germany). RNA concentration was measured using a NanoVue Plus™ spectrophotometer (Biochrom, Holliston, MA, USA). RNA purity was assessed by measuring the 260 nm/280 nm ratio. A ratio between 1.8 and 2.1 was considered as pure and high-quality RNA. Up to 3 μg RNA was reverse transcribed using the SuperScript III First-Strand Synthesis System (Thermo Fisher Scientific). Total protein concentration was determined using the Bicinchoninic Acid Protein Assay Kit (Sigma-Aldrich).

### Gene quantification by real-time polymerase chain reaction (RT-PCR)

Gene expression in animal brains and cells in culture was quantified using real-time reverse transcription polymerase chain reaction (RT-PCR). Gene expression of C-X-C motif chemokine ligand (*Cxcl*) *2*, *Cxcl10*, Interleukin (*Il*) *1b*, *Il6*, and *Tnf* was quantified by real-time RT-PCR using the gene expression assays given in Table 1.

**Table 1:** Taqman gene expression assay (Thermo Fisher Scientific) IDs

Gene	Description	Assay ID *Mus musculus*	Assay ID *Rattus norvegicus*
*Cxcl2*	C-X-C motif chemokine ligand 2	–	Rn00586403_m1
*Cxcl10*	C-X-C motif chemokine ligand 10	–	Rn01413889_g1
*Il1b*	Interleukin 1 beta	Mm00434228_m1	Rn00580432_m1
*Il6*	Interleukin 6	Mm00446190_m1	Rn01410330_m1
*Il18*	Interleukin 18	–	Rn01422083_m1
*Tnf*	Tumor necrosis factor	Mm00443258_m1	Rn01525860_g1

The PCR cycling program was run for 2 min at 50 °C, then for 10 min at 95 °C, followed by 45 cycles of 15 s at 95 °C and 1 min at 60 °C on a QuantStudio™ 7 Flex Real-Time PCR System (Thermo Fisher Scientific). The housekeeping gene glyceraldehyde-3-phosphate dehydrogenase was used as endogenous control, and primer and probe sequences were adopted from RT Primer Database [34]. Data was analyzed using the QuantStudio™ Real-Time PCR software (Thermo Fisher Scientific). Gene expression was calculated using the 2^−ΔΔCt^ method relative to untreated BV-2 or mixed glial cells or to total rat brain RNA (Amsbio, Abingdon, UK).

### Evaluation of IL-6, IL-1β, and TNFα via enzyme-linked immunosorbent assay (ELISA)

IL-1β and TNFα protein levels in brain lysates and supernatants from mixed glial cells were evaluated following the manufacturer’s instructions using rat IL-1β and TNFα DuoSet® ELISA development systems (Bio-Techne, Minneapolis, MN, USA). IL-6 and TNFα levels in supernatants from BV-2 cells were evaluated using mouse IL-6 and TNFα DuoSet® ELISA development systems (Bio-Techne) after 6 and 24 h of co-culture with exosomes.

### Intracellular signaling evaluation by Western blot analysis

Proteins were separated by sodium dodecyl sulfate polyacrylamide gel electrophoresis (SDS-PAGE) on a 4 to 20% gradient gel (Bio-Rad Laboratories, Inc., Hercules, CA, USA) and transferred onto nitrocellulose membranes (Thermo Fisher Scientific). Membranes were blocked with 5% BSA (Sigma-Aldrich) in Tris-buffered saline (TBS). Proteins were analyzed with rabbit antibodies against nuclear factor of kappa light polypeptide gene enhancer in B cells inhibitor, alpha (IκBα) (product number 9242), p44/42 MAPK (Erk1/2) (9102), phospho-p44/p42 MAPK (Erk1/2) (Thr202/Tyr204) (9101), SAPK/JNK (9252), phospho-SAPK/JNK (Thr183/Tyr185) (4668), p38 MAPK (8690), phospho-p38 MAPK (Thr180/Tyr182) (9215) (all 1:1000, Cell Signaling Technology, Boston, MA, USA), and β-tubulin (ab6046, 1:1000, Abcam) in 5% BSA in TBS-Tween 20 (Sigma-Aldrich) overnight at 4 °C. Horseradish peroxidase-coupled donkey anti-rabbit (1:1000, GE Healthcare Life Science, Piscataway, NJ, USA) antibody was used as a secondary antibody and incubated with the membranes for 1 h at room temperature. Binding was detected using the chemiluminescent Amersham ECL Prime Western Blotting Detection Reagent (GE Healthcare Life Sciences) on a Chemidoc XRS+ system (Bio-Rad). Pixel summation of individual bands was performed with ImageJ Software (NIH, Bethesda, MD, USA). The ratio between IκBα and β-tubulin as well as the ratios between the phosphorylated and the total ERK1/2, JNK, and p38 were calculated for each time point.

### Immunohistochemistry

To detect inflammation-related microglia accumulation, coronal brain paraffin sections (6 μm) were incubated with anti-ionized calcium-binding adaptor protein 1 (Iba1; 1:100; ab5076 Abcam) and anti-CD68 (1:100; ab31630 Abcam) antibodies overnight at 4 °C. Anti-Iba1 antibody was followed by a peroxidase-labeled secondary antibody (1:200; Dako, Glostrup, Denmark), and binding was visualized with diaminobenzamidine and the EnVision+ System-HRP (Dako). Anti-CD68 antibody was followed by an Alexa Fluor 488-labeled secondary antibody (1:200; Thermo Fisher Scientific). Sections were counterstained with hematoxylin (Iba1) and DAPI (CD68). Images were acquired with fluorescent (CD68) and bright field (Iba1) microscopy on a Leica DM6000 B microscope (Leica Microsystems, Wetzlar, Germany). Iba1- and CD68-positive areas within the corpus callosum were used as a measure for microglia accumulation in the white matter. Percentages of Iba1- and CD68-positive areas were quantified using ImageJ (NIH, Bethesda, MD, USA).

### Statistical analysis

We used one-way analysis of variance (ANOVA) to compare gene and cytokine expression and microgliosis between Healthy, Injury, and Injury + Exo animals. To investigate the impact of exosome treatment on gene and cytokine expression in the setting of unstimulated and LPS-stimulated cells, we used two-way ANOVA with LPS and exosome treatment as factors and also included an interaction term. One-way ANOVA was used to analyze the impact of exosome treatment on intracellular signaling. The Shapiro-Wilk test was used to test for normality of distributions in all continuous variables. Since all continuous variables were normally distributed, we applied the parametric methods as described. All reported *p* values were Bonferroni-adjusted for multiple comparison, meaning that the family-wise significance was set to 0.05. Continuous variables were expressed as mean with 95% confidence interval (CI). Differences between groups were considered significant if multiplicity-adjusted *p* values were less than 0.05. Statistical analysis was done using Prism (version 7.0, GraphPad Software, La Jolla, CA, USA).

## Results

### Characterization of hWJ-MSC and their exosomes

**Fig. 2:**
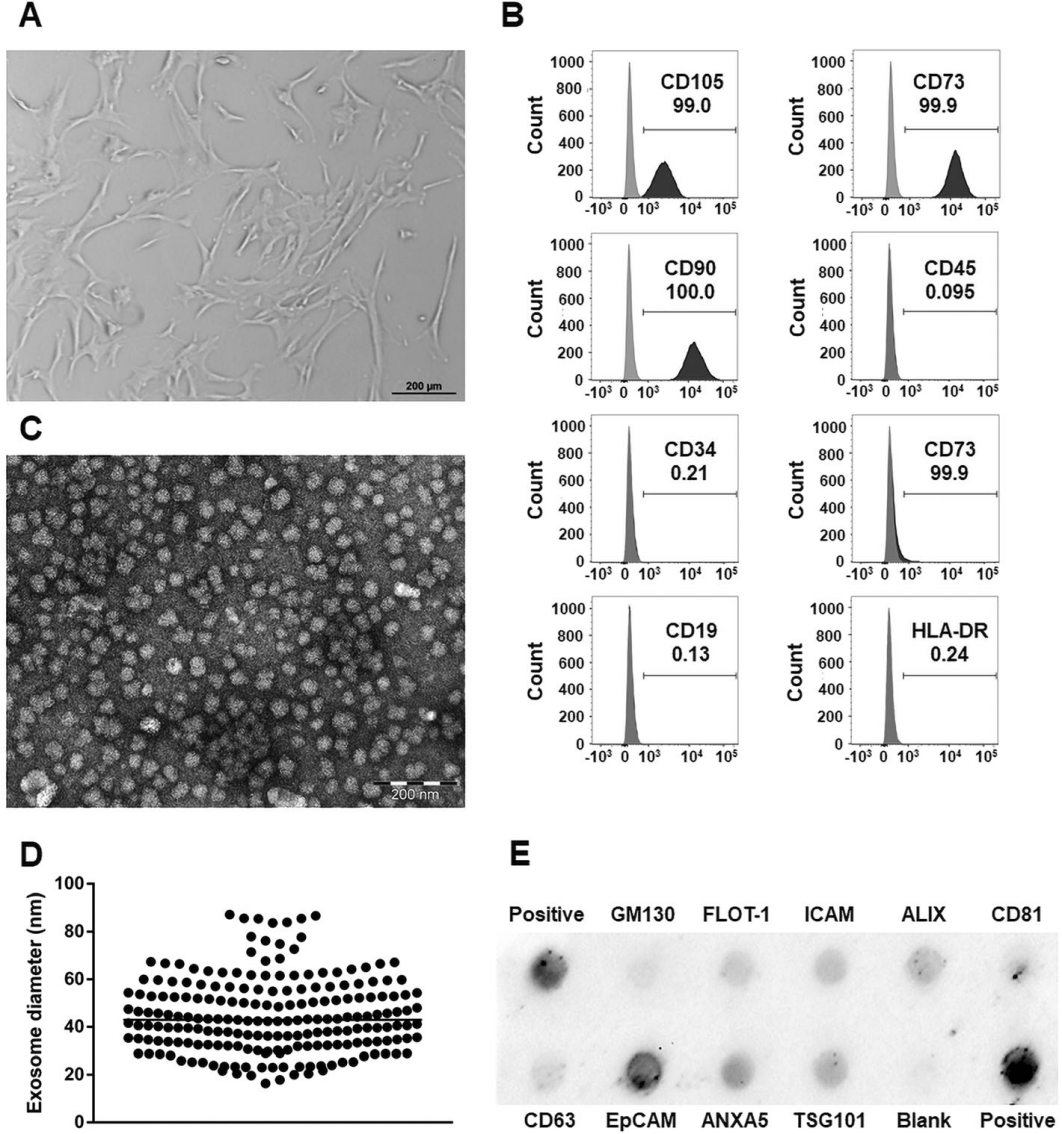
Characterization of human Wharton’s jelly mesenchymal stem cells (hWJ-MSC) and hWJ-MSC-derived exosomes. **a** Representative bright field microscopy image of hWJ-MSC. **b** Representative flow cytometry histograms of hWJ-MSC at passage 6. **c** Representative electron microscopy image of hWJ-MSC-derived exosomes (**d)** revealing a median diameter of 43 nm. **e** Representative Exo-Check antibody array of isolated exosomes

According to the minimal criteria for defining MSC by the International Society of Cellular Therapy [35], MSCs have to adhere to plastic, express classical cell surface markers, and exhibit multipotent differentiation capacity. We confirmed that the isolated hWJ-MSCs meet these criteria. Our hWJ-MSCs were plastic adherent and showed a fibroblast-like morphology (Fig. 2a). They were also highly positive for mesenchymal stem cell markers CD105, CD73, and CD90 and negative for cell differentiation markers CD45, CD34, CD14, CD19, and immunogenic marker HLA-DR (Fig. 2b). The differentiation capacity of our hWJ-MSCs into osteoblasts, chondroblasts, and adipocytes was already confirmed in our previous study [20].

Exosomes are defined as small membrane vesicles with a circular shape and a diameter of 40 to 100 nm [36]. We confirmed that the isolated exosomes are within this size range as electron microscopy revealed circular shapes (Fig. 2c) with a median diameter of 42.93 (minimum, 16.34 nm; maximum, 87.18 nm, Fig. 2d). Furthermore, exosomes should express transmembrane or lipid-bound proteins and endosomal proteins but should not express intracellular proteins associated with compartments other than plasma membrane or endosome [37]. We confirmed that the isolated exosomes expressed membrane proteins such as CD63, CD81, Flotilin 1, EpCam, and ICAM as well as endosome proteins such as TSG101, ANXA5, and ALIX but did not express cellular contamination marker cis-Golgi matrix-associated protein GM130 (Fig. 2e).

### Internalized exosomes reduce LPS-induced production of pro-inflammatory molecules in BV-2 cells

**Fig. 3:**
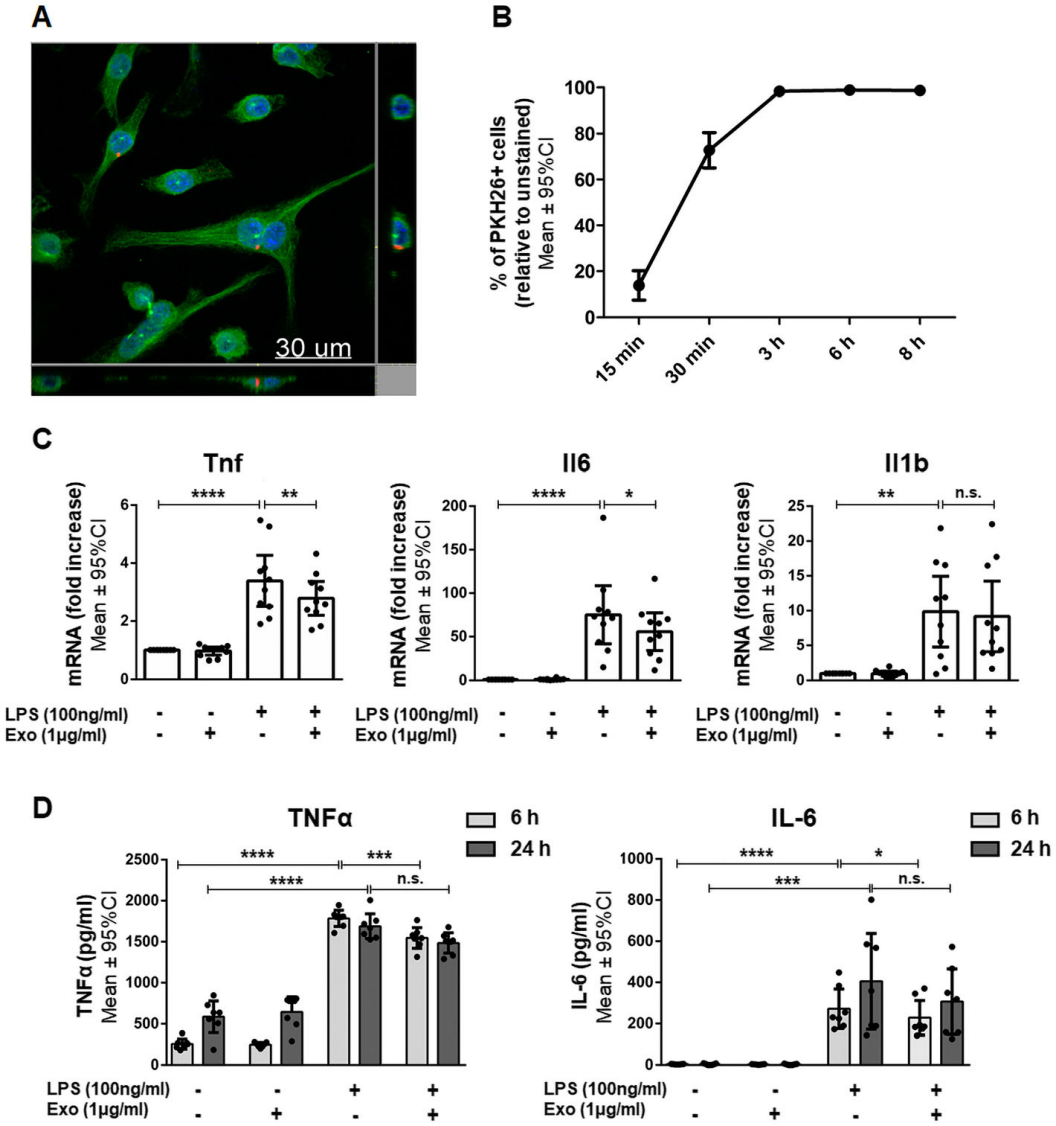
Anti-inflammatory effects of internalized human Wharton’s jelly mesenchymal stem cell-derived exosomes on BV-2 cells. **a** Representative confocal image after the co-culture of exosomes with BV-2 cells for 6 h. Exosomes were labeled with the fluorescent membrane dye PKH26 (red). BV-2 cells were stained with β-tubulin (green), and their nuclei were counterstained with 4′,6-diamidino-2′-phenylindole-dihydrochloride (DAPI) (blue). **b** PKH26-labeled exosomes (1 μg/ml) were co-cultured with BV-2 cells for 15 min, 30 min, 3 h, 6 h, or 8 h and analyzed by flow cytometry. **c** Quantification of *Tnf*, *Il6*, and *Il1b* mRNA expressed by BV-2 cells either left untreated or stimulated with LPS and/or co-incubated with 1 μg/ml exosomes for 6 h. **d** Quantification of TNFα and IL-6 secretion by BV-2 cells either left untreated or stimulated with LPS and/or co-incubated with 1 μg/ml exosomes for 6 or 24 h. Error bars illustrate mean ± 95% CI of *n* = 3 (**b**), *n* = 10 (**c**), and *n* = 7 (**d**) biological replicates. *p < 0.05 **p < 0.01, ***p < 0.001, ****p < 0.0001, *n.s.* non-significant, *CI* confidence interval, *Exo* exosomes, *Il1b/Il-1β* interleukin 1 beta, *Il6/IL-6* interleukin-6, *LPS* lipopolysaccharide, *Tnf/TNFα* tumor necrosis factor α

The interaction between hWJ-MSC-derived exosomes and their target cells is essential for the mediation of a therapeutic effect [26]. Therefore, we first tested whether hWJ-MSC-derived exosomes are taken up by murine BV-2 cells. For this, PKH26-labeled exosomes were co-cultured with BV-2 cells for 6 h. The labeled exosomes co-localized with BV-2 cells, were mainly located in the perinuclear region, and were only found within the BV-2 cell margins thus indicating their complete internalization (Fig. 3a). The internalization of PKH26-labeled exosomes by BV-2 cells was quantified using flow cytometry. For this, the cells were co-incubated with exosomes for 15 min, 30 min, 3 h, 6 h, or 8 h. Within 30 min, around 73% of BV-2 cells were PKH26-positive. After 3 h, all BV2-cells became positive for PKH26 (Fig. 3b).

The production and release of pro-inflammatory cytokines are essential in microglia-mediated inflammation. Therefore, we evaluated the capacity of hWJ-MSC-derived exosomes to prevent microglia-mediated inflammation by measuring their effect on the transcription and release of the most relevant pro-inflammatory molecules by BV-2 cells.

LPS stimulation for 6 h led to the increased transcription of genes encoding *Tnf*, *Il6*, and *Il-1β* in BV-2 cells (Fig. 3c). After 6 h of BV-2 stimulation with LPS, *Tnf* transcripts were upregulated 3.39 ± 0.88-fold (*p* < 0.0001). Exosomes suppressed *Tnf* transcript upregulation by 17.7% (2.79 ± 0.59-fold*; p* = 0.0053) compared to LPS-stimulated cells. Similarly, exosomes suppressed *Il6* transcript upregulation by 29.9% (55.71 ± 21.66-fold*; p* = 0.0267) relative to LPS-stimulated cells (75.18 ± 33.46-fold; *p* < 0.0001). Exosomes did not reduce the upregulation of *Il1b* (9.16 ± 5.08-fold; *p* > 0.9999) compared to LPS-stimulated cells (9.84 ± 5.09-fold; *p* = 0.0022). The co-culture of unstimulated BV-2 cells with exosomes did not induce any significant up- or downregulations in the expression of the assessed genes (Fig. 3c). There was a significant interaction between exposure and treatment in the sense that exosome treatment had only an impact on LPS-exposed cells.

LPS stimulation induced the secretion of pro-inflammatory cytokines TNFα and IL-6 in BV-2 cells. After 6 h of LPS stimulation, BV-2 cells secreted significantly more TNFα (1785 ± 99 pg/ml) than unstimulated cells (257 ± 61 pg/ml; *p* < 0.0001) (Fig. 3d). Exosomes dampened the LPS-induced TNFα secretion by 13.4% (1546 ± 126 pg/ml; *p* = 0.0005) compared to LPS-stimulated cells. After 24 h of LPS stimulation, BV-2 cells secreted significantly more TNFα (1689 ± 151 pg/ml) than unstimulated cells (586 ± 192 pg/ml; *p* < 0.0001). Exosomes did not decrease the LPS-induced TNFα level (1485 ± 124 pg/ml; *p* = 0.2561) compared to LPS-stimulated cells. After 6 and 24 h of LPS stimulation, BV-2 cells also secreted significantly more IL-6 (272 ± 96 pg/ml and 405 ± 232 pg/ml) than unstimulated cells (4 ± 1 pg/ml; *p* < 0.0001 and 4 ± 1 pg/ml; *p* = 0.0003) (Fig. 3d). Exosomes dampened the LPS-induced increase in IL-6 secretion significantly by 16% after 6 h (228 ± 83 pg/ml; *p* = 0.0483). After 24 h, exosomes had no effect on LPS-induced IL-6 secretion (308 ± 158 pg/ml; *p* > 0.9999). The co-culture of unstimulated BV-2 cells with exosomes did not induce any significant in- or decrease in cytokine secretion (Fig. 3d). As reported for the gene expression levels, the interaction between LPS stimulation and exosome treatment was significant; exosome treatment influenced cytokine secretion in LPS-stimulated cells only. Levels of Il-1β secretion were also measured in the cell culture supernatants, but they were below the detectable range of the used ELISA kit.

### Exosomes inhibit LPS-induced TLR4/CD14 signaling in BV-2

**Fig. 4:**
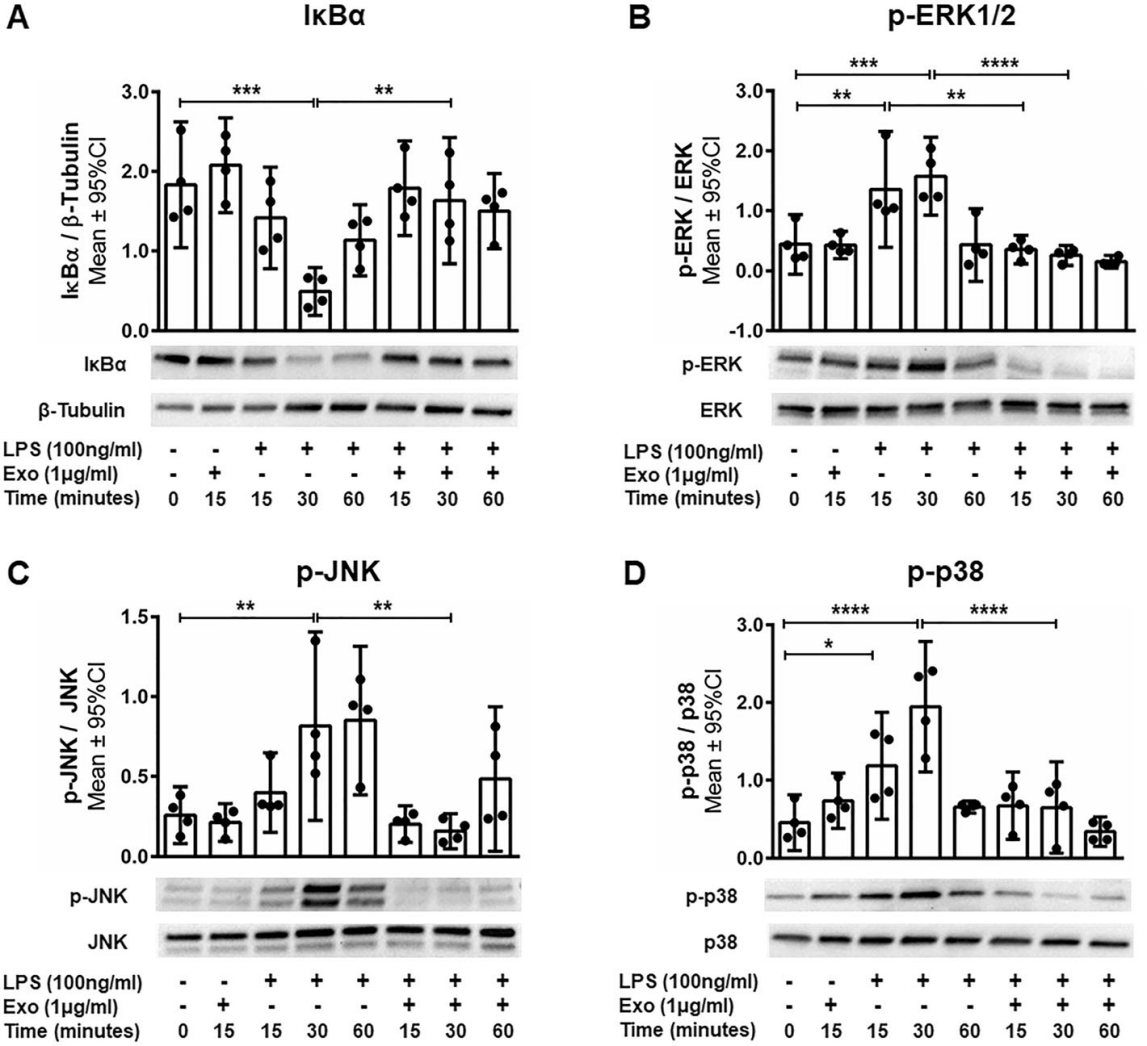
The anti-inflammatory effects of human Wharton’s jelly mesenchymal stem cell-derived exosomes are mediated via the Toll-like receptor 4 pathway. BV-2 cells were either left untreated or stimulated with LPS and/or co-incubated with exosomes for 15, 30 or 60 min. Western blot analysis of the expression of **a** NFκB inhibitor IκBα and the phosphorylation of MAPK family molecules **b** ERK, **c** JNK, and **d** p38. Error bars illustrate mean ± 95% CI of *n* = 4 biological replicates. *p < 0.05, **p < 0.01, ***p < 0.001, ****p < 0.0001 *CI* confidence interval, *Exo* exosome, *p* phosphorylation, *IκBα* nuclear factor of kappa light polypeptide gene enhancer in B cells inhibitor alpha *MAPK* mitogen-activated protein kinase, *LPS* lipopolysaccharide, *NFκB* nuclear factor kappa-light-chain-enhancer of activated B cells

LPS stimulates microglia via the membrane receptor complex TLR4/CD14, inducing the production of pro-inflammatory cytokines such as TNFα, IL-6, and interferon gamma (IFNγ). The TLR4/CD14 signaling pathway is mediated by regulators of NFκB such as IκBα, as well as by regulators of activation protein 1 (AP-1) such as the mitogen-activated protein kinase (MAPK) family members ERK1/2, p38, and JNK. We assessed whether hWJ-MSC-derived exosomes interfere with the degradation of IκBα and the phosphorylation of the MAPK family to prevent NFκB- and AP-1-dependent transcription of pro-inflammatory cytokines. The degradation of IκBα was significantly prevented after 30 min of LPS stimulation in BV-2 cells co-cultured with exosomes compared to LPS-stimulated BV-2 cells cultured without exosomes (*p* < 0.01, Fig. 4a). Also, the exosomes significantly decreased the phosphorylation of ERK1/2 (*p* < 0.01) after 15 min and of ERK1/2 (*p* < 0.0001), JNK (*p* < 0.01), and p38 (*p* < 0.0001) after 30 min of LPS stimulation compared to the LPS-stimulated BV-2 cells in absence of exosomes (Fig. 4b–d). After 60 min, no difference in IκBα expression and ERK1/2, p38, and JNK phosphorylation between LPS-stimulated BV-2 cells cultured in the presence and absence of exosomes and unstimulated cells could be observed (Fig. 4).

### Internalized exosomes reduce LPS-induced production of pro-inflammatory molecules in primary mixed glial cells

Even though BV-2 cells are widely accepted as an appropriate cell line model for murine microglia, they do not reflect all properties of primary microglia. Thus, we aimed to confirm the immunomodulatory effects of hWJ-MSC-derived exosomes found in BV-2 cells using primary mixed glial cells consisting of microglia and astrocytes.

**Fig. 5:**
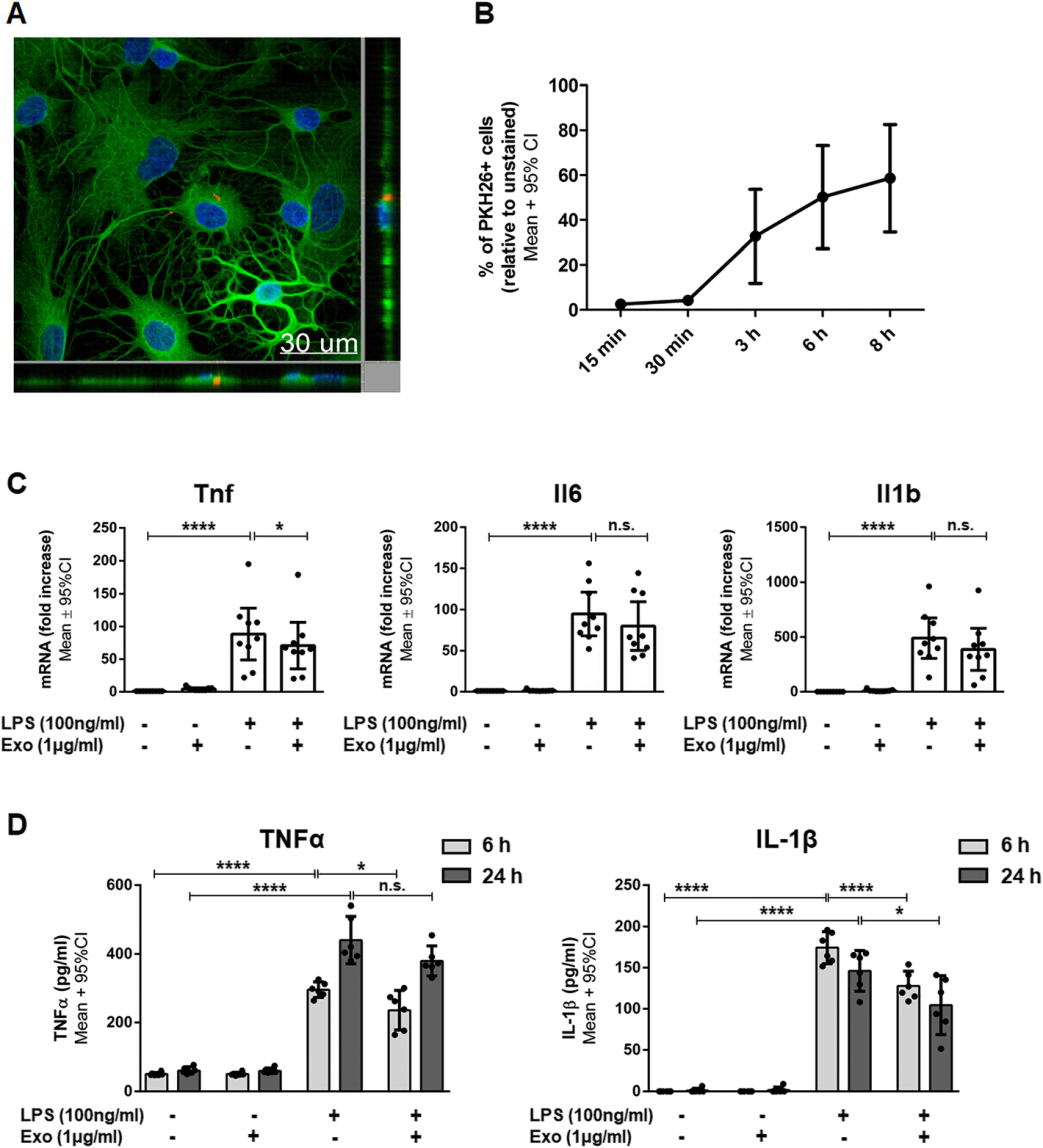
Anti-inflammatory effects of internalized human Wharton’s jelly mesenchymal stem cell-derived exosomes on primary mixed glial cells. **a** Representative confocal image after the co-culture of exosomes with primary mixed glial cells for 6 h. Exosomes were labeled with the fluorescent membrane dye PKH26 (red). Primary mixed glial cells were stained with β-tubulin (green) and their nuclei were counterstained with 4′,6-diamidino-2′-phenylindole-dihydrochloride (DAPI) (blue). **b** PKH26-labeled exosomes (1 μg/ml) were co-cultured with mixed glial cells for 15 min, 30 min, 3 h, 6 h, or 8 h and analyzed by flow cytometry. **c** Quantification of *Tnf*, *Il6*, and *Il1b* mRNA expressed by mixed glial cells either left untreated or stimulated with LPS and/or co-incubated with exosomes for 6 h. **d** Quantification of TNFα and IL-1β secretion by mixed glial cells either left untreated or stimulated with LPS and/or co-incubated with exosomes for 6 or 24 h. Error bars illustrate mean ± 95% CI of *n* = 3 (**b**), *n* = 9 (**c**), and *n* = 6 (**d**) biological replicates. *p < 0.05, ****p < 0.0001, *n.s.* non-significant, *CI* confidence interval, *Exo* exosomes, *Il1b/Il-1β* interleukin 1 beta, *Il6/IL-6* interleukin-6, *LPS* lipopolysaccharide, *Tnf/TNFα* tumor necrosis factor α

Again, we tested whether the exosomes are taken up by rat mixed glial cells. After 6 h of co-culture, the PKH26-labeled exosomes co-localized with mixed glial cells, were mainly located in the perinuclear region, and were only found within the mixed glial cell margins, thus indicating their complete internalization (Fig. 5a). As previously done for BV-2 cells, we quantified exosome internalization by mixed glial cells using flow cytometry. For this, mixed glial cells were co-incubated with PKH26-labeled exosomes. After 3 h of co-culture, 30% of mixed glial cells became positive for PKH26. Upon 8 h of co-incubation, nearly 60% of cells were PKH26 positive (Fig. 5b).

Comparable to what we found in BV-2 cells, the transcription of the genes encoding TNFα, IL-6, and IL-1β was upregulated after LPS stimulation in mixed glial cells. Furthermore, exosomes dampened the transcription of *Tnf*, but not the transcription of *Il6* and *Il1b* in mixed glial cells stimulated with LPS (Fig. 5c). Specifically, after 6 h of LPS stimulation of mixed glial cells, *Tnf* transcripts were upregulated 88.40 ± 39.62-fold (*p* < 0.0001). Exosomes suppressed *Tnf* transcripts by 20.1% (70.68 ± 35.55-fold; *p* = 0.0168) compared to LPS-stimulated cells. Exosomes did not influence the LPS-stimulated upregulation of *Il6* and *Il1b* transcription (80.04 ± 29.62-fold; *p* > 0.999 and 386.88 ± 192.39-fold; *p* > 0.999) compared to LPS-stimulated mixed glial cells (94.73 ± 26.58-fold; *p* < 0.0001 and 490.04 ± 185.22-fold; *p* < 0.0001). The co-culture of unstimulated mixed glial cells with exosomes did not induce any significant up- or downregulations in the expression of the assessed genes (Fig. 5c). The interaction between exposure and treatment was significant, and exosome treatment had only an impact on LPS-exposed cells.

At the protein level, similar to our findings in BV-2 cells, stimulation with LPS led to a statistically significant increase in TNFα and IL-1β secretion in mixed glial cells. Additionally, exosomes significantly dampened the secretion of both TNFα and IL-1β by mixed glial cells after LPS stimulation (Fig. 5d). After 6 h of LPS stimulation, mixed glial cells secreted significantly more TNFα (295.90 ± 22.86 pg/ml) than unstimulated cells (50.83 ± 4.67 pg/ml; *p* < 0.0001). Exosomes dampened the LPS-induced TNFα secretion by 20.3% (235.96 ± 57.61 pg/ml; *p* = 0.0137) relative to LPS-stimulated cells. After 24 h of LPS stimulation, mixed glial cells still secreted significantly more TNFα (440.83 ± 68.96 pg/ml) than unstimulated cells (60.99 ± 9.60 pg/ml; *p* < 0.0001). Exosomes did not affect the TNFα secretion in stimulated cells (379.92 ± 43.64 pg/ml; *p* > 0.999). After 6 h of LPS stimulation, mixed glial cells also secreted significantly more IL-1β (174.39 ± 19.53 pg/ml) than unstimulated cells (< 62.5 pg/ml [values below detectable range]; *p* < 0.0001). In stimulated mixed glia cells, exosomes significantly reduced the IL-1β secretion by 26.7% (127.79 ± 17.81 pg/ml; *p* < 0.0001) relative to LPS-stimulated cells. After 24 h of LPS stimulation, mixed glial cells still secreted significantly more IL-1β (146.09 ± 24.78 pg/ml) than unstimulated cells (1.08 ± 0.62 pg/ml; *p* < 0.0001) and exosomes significantly dampened the upregulation of IL-1β secretion by 28.5% (104.47 ± 35.93 pg/ml; *p* = 0.0153) compared to LPS-stimulated cells. The co-culture of unstimulated mixed glial cells with exosomes did not induce any significant change in cytokine secretion. There was a significant interaction between exposure and treatment since exosome treatment had an impact on LPS-exposed cells only.

### hWJ-MSC-derived exosomes inhibit the production of pro-inflammatory molecules and prevent microgliosis in rats with perinatal brain injury

**Fig. 6:**
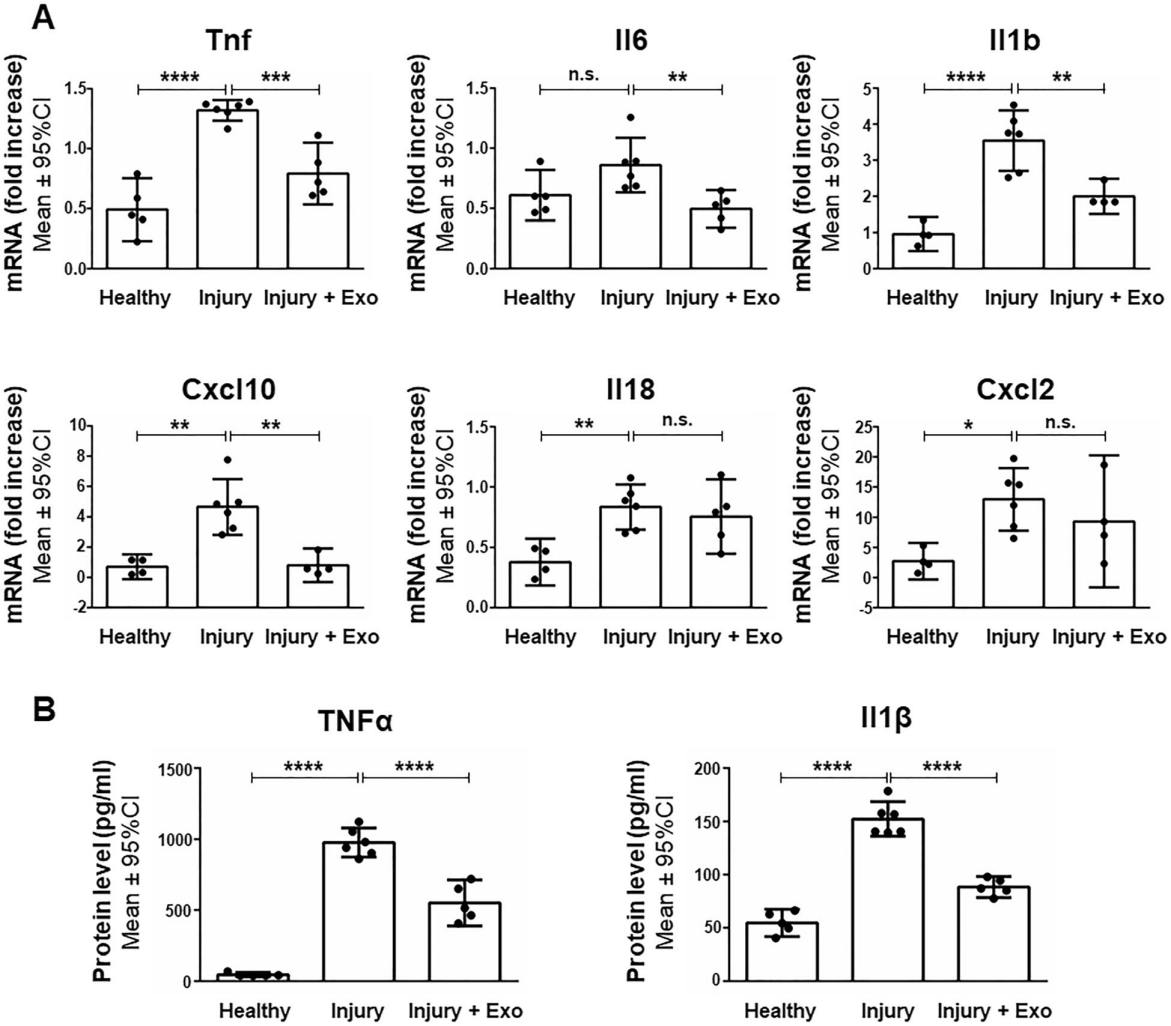
Anti-inflammatory effects of human Wharton’s jelly mesenchymal stem cell-derived exosomes in perinatal brain injury. **a** Quantification of *Tnf*, *Il6*, *Il1b*, *Cxcl10*, *Il18*, and *Cxcl2* mRNA expressed in the brain of healthy rats and of rats 24 h after injury with and without exosome treatment. **b** Quantification of TNFα and IL-1β expression in the brain parenchyma of healthy rats and of rats 24 h after brain injury with and without exosome treatment. Error bars illustrate mean ± 95% CI of at least four different animals. *p < 0.05, **p < 0.01 ***p < 0.001, ****p < 0.0001, *n.s.* non-significant, *CI* confidence interval, *Cxcl2* C-X-C motif chemokine ligand 2, *Cxcl10* C-X-C motif chemokine ligand 10, *Exo* exosomes, *Il1b/Il-1β* interleukin 1 beta, *Il18* interleukin 18, *Il6* interleukin-6, *Tnf/TNFα* tumor necrosis factor alpha

Next, we evaluated the anti-inflammatory potential of intranasally administered hWJ-MSC-derived exosomes in a rat model of perinatal brain injury. The expression of genes encoding pro-inflammatory markers (*Cxcl2*, *p* = 0.0177; *Cxcl10*, *p* = 0.0010; *Il1b*, *p* < 0.0001; *Il18*, *p* = 0.0066; *Tnf*, *p* < 0.0001) was significantly increased in the brain 24 h after brain injury. Intranasal administration of exosomes significantly prevented this upregulation for most pro-inflammatory markers analyzed (*Cxcl10*, *p* = 0.0012; *Il1b*, *p* = 0.0034; *Il6*, *p* = 0.0099; *Tnf*, *p* = 0.0004) relative to untreated animals (Fig. 6a). The protein expression of pro-inflammatory cytokines such as IL-1β and TNFα in brain parenchyma was strongly upregulated 24 h after brain injury. Intranasal administration of exosomes significantly prevented the increased expression of these cytokines. More specifically, LPS stimulation induced a 20-fold expression of TNFα 24 h after brain injury (976.82 ± 102.32 pg/ml) relative to healthy animals (46.23 ± 15.37 pg/ml; *p* < 0.0001). Exosomes successfully decreased the injury-related TNFα expression by 43.6% (550.86 ± 162.54 pg/ml; *p* < 0.0001). IL-1β secretion was three-fold higher 24 h after brain injury (152.32 ± 16.04 pg/ml) than in healthy animals (54.62 ± 12.80 pg/ml; *p* < 0.0001). Exosomes decreased the IL-1β expression in injured brains by 42.0% (88.40 ± 9.93 pg/ml; *p* < 0.0001) (Fig. 6b).

**Fig. 7:**
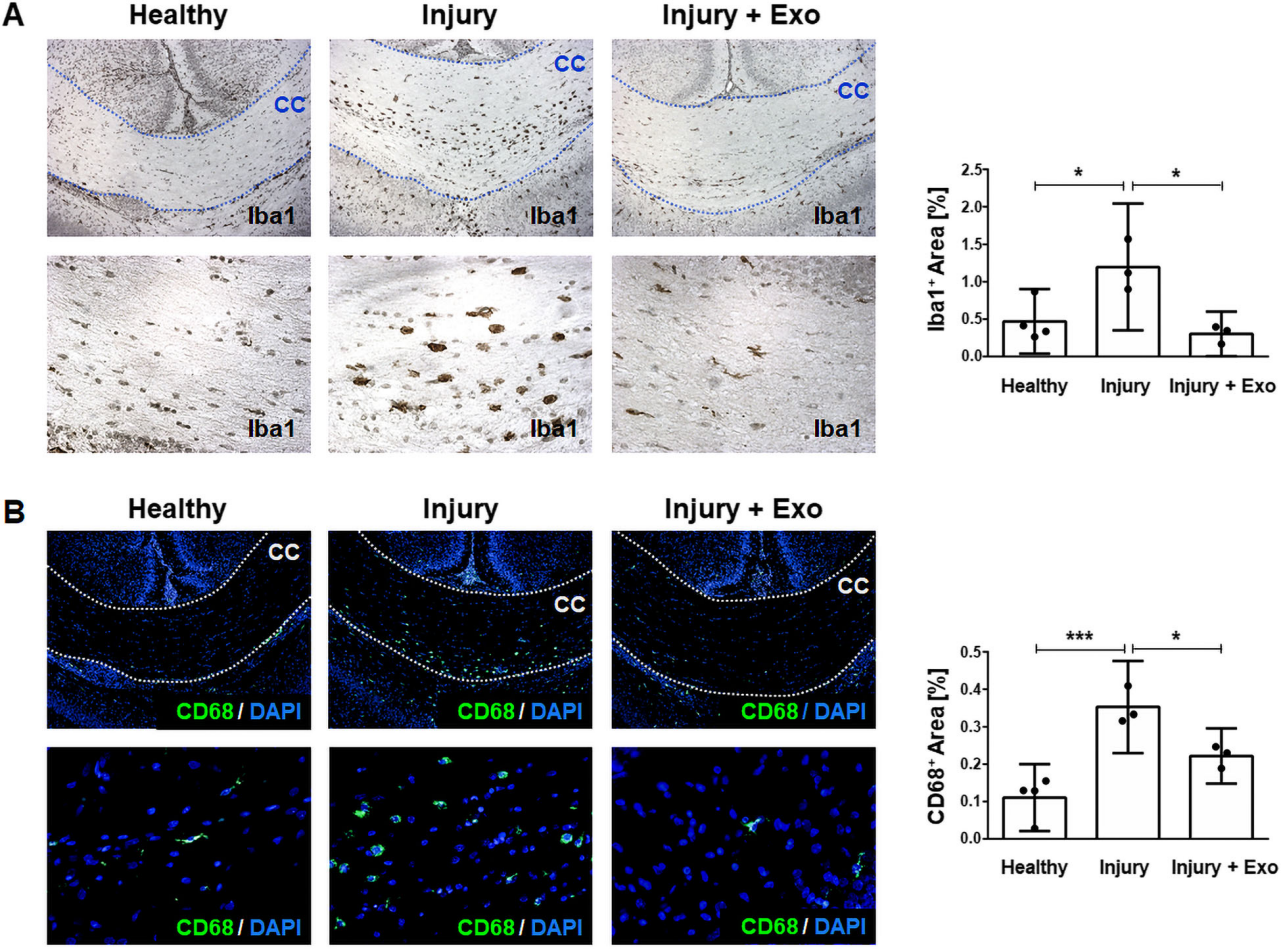
Prevention of microgliosis by human Wharton’s jelly mesenchymal stem cell-derived exosomes in perinatal brain injury. **a** Representative chromogenic immunohistochemistry images and quantification of Iba1^+^ cells in the corpus callosum of healthy rats and of rats 24 h post brain injury with and without exosome treatment. **b** Representative fluorescent immunohistochemistry images and quantification of CD68^+^ cells in the corpus callosum of healthy rats and of rats 24 h after brain injury with and without exosome treatment. Bottom row images are higher magnifications (40x) of the top row images (10x). Error bars illustrate mean ± 95% CI of at least three different animals. *p < 0.05, ***p < 0.001, *CC* corpus callosum, *CI* confidence interval, *CD68* cluster of differentiation 68, *DAPI* 4′,6-diamidino-2′-phenylindole-dihydrochloride, *Exo* exosomes, *Iba1* ionized calcium-binding adaptor molecule

A key hallmark of the brain’s response to insults is the activation and proliferation of microglia, a reaction called microgliosis. Thus, we analyzed the potential of hWJ-MSC-derived exosomes to prevent microgliosis in the corpus callosum, a white matter region known to be especially vulnerable to perinatal brain injury [38, 39]. In healthy animals, very few microglia are present within the corpus callosum as only 0.47 ± 0.43% and 0.11 ± 0.09% of the total area of the corpus callosum stained positive for microglia markers Iba1 (Fig. 7a) and CD68 (Fig. 7b), respectively. One day after injury, the area of the corpus callosum occupied by Iba1^+^ cells was increased by 60.7% (1.20 ± 0.81% of the total area; *p* = 0.0256). Upon treatment of injured animals with exosomes, the region of Iba1^+^ cells decreased by 75% and reached baseline values (0.30 ± 0.29% of total area; *p* = 0.0126). Similarly, the area occupied by CD68^+^ cells increased in the first 24 h after perinatal brain injury (0.35 ± 0.12% of total area; *p* = 0.0009). Exosome treatment reduced the area occupied by CD68^+^ cells in injured brains by 37% (0.22 ± 0.07% of total area; *p* = 0.0379). Following perinatal brain injury, no microgliosis could be detected in areas of the cortex, thalamus, and hippocampus (see Additional file 1: Figure S1).

## Discussion

Our study revealed that hWJ-MSC-derived exosomes have anti-inflammatory effects in perinatal brain injury. In vitro, we demonstrated that the exosomes are internalized by microglia cells and reduce the release of pro-inflammatory cytokines by microglial cells in response to LPS stimulation. In vivo, we showed that intranasal administration of hWJ-MSC-derived exosomes is an effective treatment to reduce neuroinflammation.

The uptake of hWJ-MSC-derived exosomes into their target cells is an essential prerequisite for their therapeutic action [26]. In vitro, we showed that hWJ-MSC-derived exosomes are very efficiently internalized by BV-2 microglia and to a slightly less efficient extent into primary mixed glial cells. We propose that this difference in uptake efficiency is due to the different compositions of these two cell types. While the immortalized BV-2 cells consist purely of microglia cells, the primary mixed glial cells consist of a mixture of astrocytes and microglia cells. Astrocytes have been described as much less efficient as microglia in clearing debris [40] and specifically in internalizing exosomes [41, 42]. Hence, it is likely that most of the mixed glial cells that internalized exosomes in our experiment were microglia cells, although this hypothesis remains to be confirmed by further experiments. Within the brain, microglia cells have been previously described to be the primary cell type to internalize exosomes after their intranasal administration in mice [43]. Whether this also holds true in rats after perinatal brain injury remains to be tested in future experiments.

Microglia play a pivotal role in the pathophysiology of perinatal brain injury. Microglial cells exhibit dualistic functions and can be categorized into a pro-inflammatory M1 and an anti-inflammatory, regenerative M2 phenotype [44]. In perinatal brain injury, microglia initially respond to stimuli such as hypoxia-ischemia or infection with the production of pro-inflammatory cytokines that eventually exacerbate brain injury. After the initial pro-inflammatory phase, microglia eventually switch their phenotype and start to produce anti-inflammatory molecules favoring tissue repair and neuroregeneration [13]. A complete abrogation of microglia function could therefore have detrimental effects in perinatal brain injury. This dual functionality of microglia makes immunomodulation the most promising approach for optimizing the response of microglial cells during neuroinflammation. Here, we showed that hWJ-MSC-derived exosomes modulate the microglial response by interfering with the TLR4/CD14 signaling pathway. Given that exosomes are well known for their capacity to deliver micro RNAs (miRNAs) [26, 36], we hypothesize that exosomal miRNAs are responsible for the observed immunomodulation. We have previously described the miRNA content of hWJ-MSC-derived exosomes [32]. Several exosomal miRNAs have been described to be negative regulators of inflammatory gene expression, such as miR-146a/b, which negatively regulate the production of pro-inflammatory cytokines via TLR4/CD14 signaling [45]. Further analysis of MSC-derived exosomes is required to better understand how these miRNAs may convey anti-inflammatory and immunomodulatory signals to microglia.

Specific interference with TLR4/CD14 signaling explains why hWJ-MSC-derived exosomes, at least in vitro, are only effective in dampening certain pro-inflammatory cytokines such as IL-6 and TNFα and not others such as Il-1β and IL-18. While IL-6 and TNFα are direct target genes of the TLR4/CD14 pathway, IL-18 and IL-1β are targets of the inflammasome cascade [46]. hWJ-MSC-derived exosomes seem to be effective at interfering with TLR4/CD14 signaling but not at interfering with the inflammasome cascade in vitro. This finding stands in contrast to the effects of hWJ-MSC-derived exosomes in our animal model, where they seem to be able to inhibit IL-1β (but not IL-18), indicating that hWJ-MSC-derived exosomes at least partially interfere with the inflammasome cascade in vivo. The specific effects of hWJ-MSC-derived exosomes on the inflammasome cascade and on further pathways in animal models are to be confirmed.

Furthermore, hWJ-MSC-derived exosomes may reduce neuroinflammation in vivo in part through peripheral immunomodulation. After intranasal administration, some exosomes end up in peripheral organs such as the spleen. Recently, MSC-derived exosomes have been shown to modulate peripheral immune responses [47] which prevented subsequent immune cell invasion into the brain and thus reduced brain injury [48]. We suggest that hWJ-MSC-derived exosomes not only have direct immunomodulatory effects on microglia in the central nervous system, but also have additional immunomodulatory effects in the periphery. These additional immunomodulatory effects outside of the brain could explain why we observed substantially higher immunomodulatory effects in our animal model than in the microglial cell cultures. This possibility highlights the importance of further research into the immunomodulatory effects of MSC-derived exosomes in the periphery.

Microglia-mediated inflammation in our animal model of preterm brain injury seems to occur predominantly in the corpus callosum, the main white matter structure in the brain. This is in accordance with studies showing that cerebral white matter regions are especially injury-prone in infants born prematurely [38, 39]. The selective vulnerability to white matter injury is closely related to the fetal brain development. During 23 to 32 weeks of gestation, oligodendrocyte progenitor cells develop according to a well-defined sequence of maturational events. Hypoxic-ischemic or inflammatory insults in infants born very prematurely (< 32 weeks of gestation) cause oligodendrocyte progenitor cells to arrest their maturation which results in white matter injury [49, 50].

Our results implicating an anti-inflammatory function of hWJ-MSC-derived exosomes are in line with previous studies documenting the repressive effect of MSC and their derived extracellular vesicles on microglial activation. Supporting our observations, it was recently demonstrated that extracellular vesicles retrieved from human bone marrow MSC reduced microgliosis in a rat model of inflammation-induced preterm brain injury [51]. Moreover, exosomes derived from exfoliated deciduous teeth stem cells have also been reported to inhibit IL-6 and TNFα production in LPS-stimulated BV-2 microglial cells and to dampen microglia activation in an animal model of traumatic brain injury [9]. Similar effects were observed in a transwell co-culture system with mouse bone marrow MSC and BV-2 cells. Upon LPS stimulation, the presence of MSC in the co-culture decreased TNFα expression but increased IL-6 expression in a cell dose-dependent manner [12]. While the decreased TNFα expression is in line with our study, the increased IL-6 level is not. This discrepancy can partially be explained by the fact that in the aforementioned study, the IL-6 was not only produced by BV-2 cells but also by MSC [12]. Interestingly, immunomodulatory capacities comparable to the ones of exosomes were also described for bone marrow MSC-derived microvesicles (MV) [10]. MSC-MV were able to decrease pro-inflammatory cytokines such as IL-6 and TNFα and to inhibit the phosphorylation of the MAPK family molecules in the TLR4/CD14 pathway in LPS-stimulated BV-2 cells [10].

Our findings further support studies investigating the therapeutic potential of MSC-derived exosomes in preterm-associated diseases. hWJ-MSC-derived exosomes have been successfully used to treat bronchopulmonary dysplasia [29] and hypoxia-induced pulmonary hypertension [52] in newborn mice. Similar to our results, hWJ-MSC-derived exosomes dampened hyperoxia-induced inflammation by decreasing the transcription of *Il6* and *Tnf* in alveolar macrophages [29] and prevented hypoxia-induced macrophage infiltration into the lungs [52]. Exosomes derived from bone marrow MSC have also been proven to be an effective treatment for necrotizing enterocolitis as they reduced the incidence and the extent of the disease in premature newborn rats [28].

Stem cell-based therapies hold great clinical potential when it comes to treating inflammatory diseases. Stem cells derived from the tissue of the umbilical cord can be easily obtained, as umbilical cords are readily available and otherwise regarded as a biological waste product. Umbilical cord stem cells can be used as an autologous graft which substantially reduces the risk of a potential transplant rejection. Both stem cells [14] and their exosomes can be intranasally delivered to the brain and this non-invasive route of application makes the therapy especially suitable for translation into clinical practice. Although the intranasal administration of MSC has been shown to be effective in perinatal brain injury [14], the use of MSC-derived cell-free vesicles has several additional advantages. For example, it is unavoidable that upon transplantation into an injured brain, MSC, in contrast to MSC-derived exosomes, respond to the inflammatory stimuli by producing further pro-inflammatory molecules [11]. Additionally, MSC have the potential to get trapped in the lungs [53] and to induce tumor growth in transplanted tissue [54, 55]. These risks can be easily eliminated by using MSC-derived exosomes. Furthermore, exosomes are easier to store compared to MSC as they can be kept suspended in PBS at − 20 °C. However, the use of stem cell-derived vesicles as a treatment creates new challenges in terms of their standardized isolation, quantification, characterization, and storage as well as in terms of their route and timing of administration [56]. In this study, we administered hWJ-MSC-derived exosomes at the same time as the injury was introduced. By choosing such an early time point of treatment, we were able to demonstrate a strong anti-inflammatory effect of the exosomes in the acute phase of the injury. Future research will focus on evaluating the exosomes as a post-injury treatment to increase their clinical relevance. Finding the optimal time point(s) of administration and dosage are major challenges in the standardization of vesicle therapies and remain to be solved before such treatments will make their way into the clinics.

## Conclusions

In conclusion, hWJ-MSC-derived exosomes have anti-inflammatory effects on microglia in perinatal brain injury. These anti-inflammatory effects are at least in part caused by exosome-mediated interference with the TLR4/CD14 signaling cascade, thereby leading to a dampened transcription of inflammation-related genes. In the brain, exosomes reduce neuroinflammation by suppressing the transcription and secretion of pro-inflammatory cytokines and inhibiting the accumulation of microglia. This mechanism prevents an exacerbated inflammatory reaction, which is important to avoid secondary brain injury leading to subsequent neurological deficits. Hence, hWJ-MSC-derived exosomes represent a promising future cell-free therapy to prevent and treat perinatal brain injury in preterm infants.

## Additional file

Additional file 1: **Figure S1.** Microgliosis in gray matter areas after perinatal brain injury (PPTX 5412 kb)

## Abbreviations

ALIX: ALG-2-interacting protein X
ANXA5: Annexin A5
BSA: Bovine serum albumin
CD: Cluster of differentiation
Cxcl10: C-X-C motif chemokine ligand 10
Cxcl2: C-X-C motif chemokine ligand 2
DAPI: 4′,6-Diamidino-2′-phenylindole-dihydrochloride
DMEM: Dulbecco’s modified Eagle’s medium
ELISA: Enzyme-linked immunosorbent assay
EpCAM: Epithelial cell adhesion molecule
Exo: Exosomes
FBS: Fetal bovine serum
FLOT1: Flotillin 1
GM130: Cis-Golgi matrix protein 130
HLA-DR: Human leukocyte antigen–antigen D related
hWJ-MSC: Human Wharton’s jelly-derived mesenchymal stem cells
Iba1: Ionized calcium-binding adaptor protein 1
ICAM1: Intercellular adhesion molecule 1
IFNγ: Interferon gamma
*Il18*/IL-18: Interleukin-18
*Il1b*/IL-1β: Interleukin 1 beta
*Il6*/IL-6: Interleukin 6
IκBα: Nuclear factor of kappa light polypeptide gene enhancer in B cells inhibitor alpha
LPS: Lipopolysaccharide
MAPK: Mitogen-activated protein kinase
miRNA: MicroRNA
MSC: Mesenchymal stem cells
MV: Microvesicles
NFκB: Nuclear factor kappa-light-chain-enhancer of activated B cells
PBS: Phosphate-buffered saline
RT-PCR: Reverse transcription polymerase chain reaction
SDS-PAGE: Sodium dodecyl sulfate polyacrylamide gel electrophoresis
TBS: Tris-buffered saline
TLR4: Toll-like receptor 4
*Tnf*/TNFα: Tumor necrosis factor alpha
TSG101: Tumor susceptibility gene 101
