# Correction: Exosomes derived from umbilical cord mesenchymal stem cells reduce microglia-mediated neuroinflammation in perinatal brain injury

**DOI:** 10.1186/s13287-022-03079-5

**Published:** 2022-07-27

**Authors:** Gierin Thomi, Daniel Surbek, Valérie Haesler, Marianne Joerger-Messerli, Andreina Schoeberlein

**Affiliations:** 1 Department of Obstetrics and Feto-maternal Medicine, University Women’s Hospital, Inselspital, Bern University Hospital, Bern, Switzerland; 2 Department for BioMedical Research (DBMR), University of Bern, Bern, Switzerland; 3 Graduate School for Cellular and Biomedical Sciences, University of Bern, Bern, Switzerland

## Correction to: Stem Cell Research & Therapy (2019) 10:105 10.1186/s13287-019-1207-z

The original article contains an error in Fig. 2B whereby the sub-panel in column 2, row 3 is incorrect. The corrected figure can be viewed ahead.

**Fig. 2:**
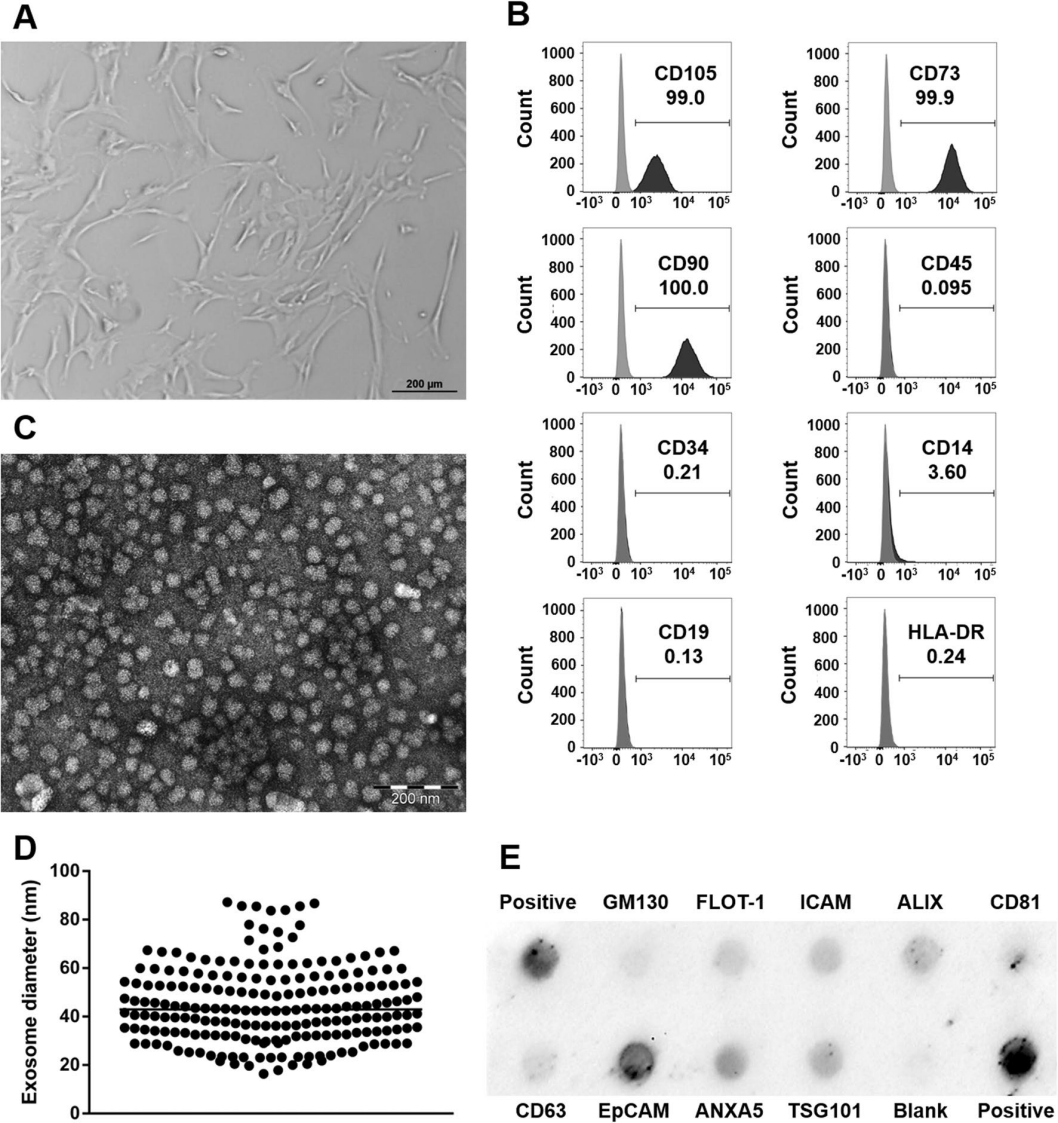
Characterization of human Wharton’s jelly mesenchymal stem cells (hWJ-MSC) and hWJ-MSC-derived exosomes. **a** Representative bright field microscopy image of hWJ-MSC. **b** Representative flow cytometry histograms of hWJ-MSC at passage 6. **c** Representative electron microscopy image of hWJ-MSC-derived exosomes (**d**) revealing a median diameter of 43 nm. **e** Representative Exo-Check antibody array of isolated exosomes

