# Supplementary material for: Exosomes derived from umbilical cord mesenchymal stem cells reduce microglia-mediated neuroinflammation in perinatal brain injury
Source: Stem Cell Res Ther. 2019 Mar 21;10:105. doi: 10.1186/s13287-019-1207-z (PMC6429800; doi:10.1186/s13287-019-1207-z)
Supplement: Supplementary file 1 — Figure S1. Microgliosis in gray matter areas after perinatal brain injury (PPTX 5412 kb) [file 13287_2019_1207_MOESM1_ESM.pptx]

## Slide 1
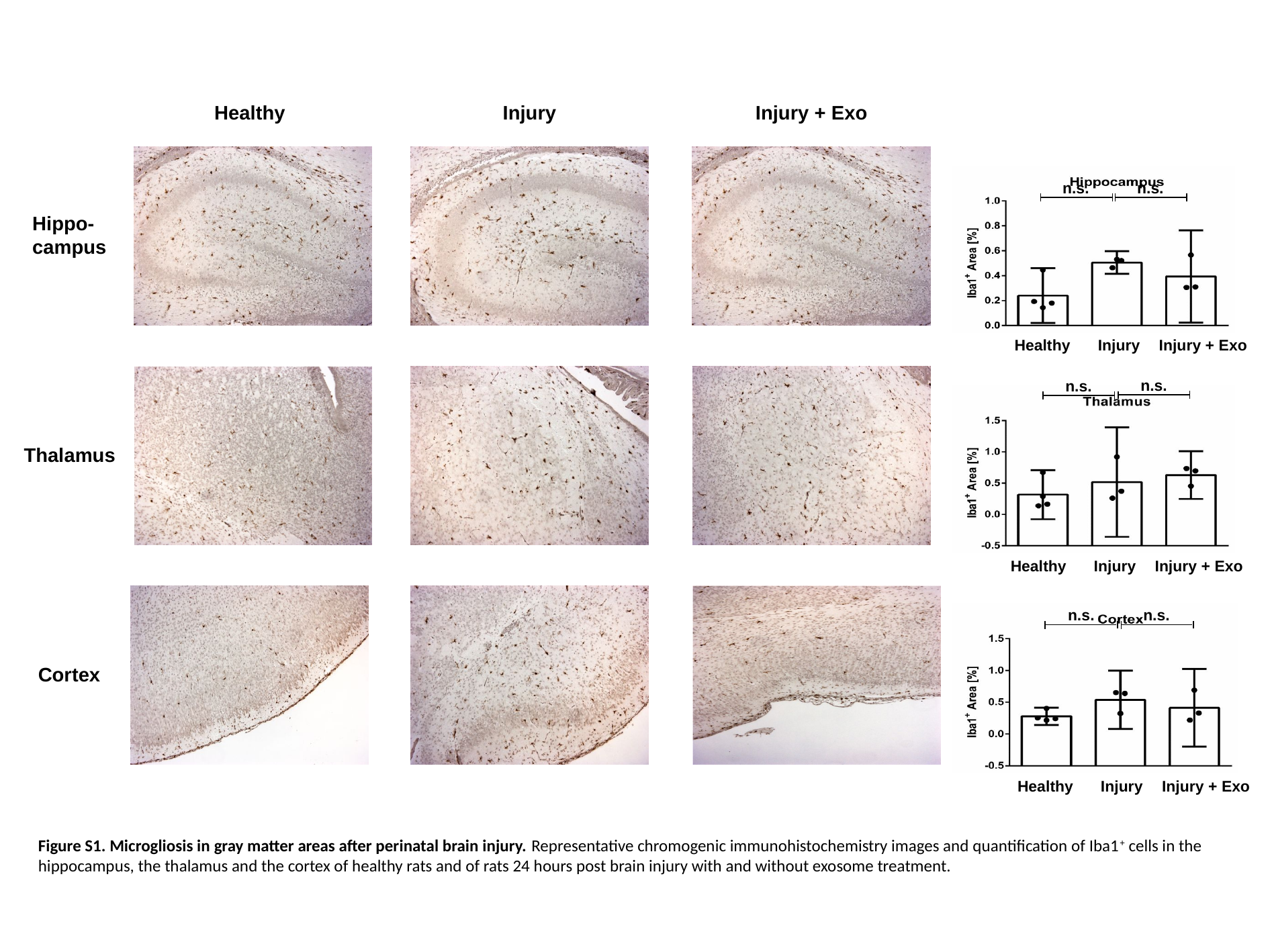

Healthy
Injury
Injury + Exo
n.s.
n.s.
Hippo-campus
Healthy
Injury
Injury + Exo
n.s.
n.s.
Thalamus
Healthy
Injury
Injury + Exo
n.s.
n.s.
Cortex
Healthy
Injury
Injury + Exo
Figure S1. Microgliosis in gray matter areas after perinatal brain injury. Representative chromogenic immunohistochemistry images and quantification of Iba1+ cells in the hippocampus, the thalamus and the cortex of healthy rats and of rats 24 hours post brain injury with and without exosome treatment.
